# Long-term association of pregnancy and maternal brain structure: the Rotterdam Study

**DOI:** 10.1007/s10654-021-00818-5

**Published:** 2022-01-06

**Authors:** Jurate Aleknaviciute, Tavia E. Evans, Elif Aribas, Merel W. de Vries, Eric A. P. Steegers, Mohammad Arfan Ikram, Henning Tiemeier, Maryam Kavousi, Meike W. Vernooij, Steven A. Kushner

**Affiliations:** 1 Department of Psychiatry, Erasmus MC, University Medical Center Rotterdam, ‘s Gravendijkwal 230, 3000 CA Rotterdam, The Netherlands; 2 Department of Clinical Genetics, Erasmus MC, University Medical Center Rotterdam, Wytemaweg 90, 3015 CN Rotterdam, The Netherlands; 3 Department of Radiology, Erasmus MC, University Medical Center Rotterdam, Wytemaweg 80, 3015 CN Rotterdam, The Netherlands; 4 Department of Epidemiology, Erasmus MC, University Medical Center Rotterdam, Wytemaweg 90, 3015 CN Rotterdam, The Netherlands; 5 Department of Obstetrics and Gynecology, Erasmus MC-Sophia Children’s Hospital, University Medical Center Rotterdam, Wytemaweg 80, 3015 CN Rotterdam, The Netherlands; 6 Department of Social and Behavioral Sciences, Harvard T.H. Chan School of Public Health, Boston, MA USA; 7 Department of Child Psychiatry, Sophia Children’s Hospital, Erasmus University Medical Center, Wytemaweg 80, 3015 CN Rotterdam, The Netherlands

**Keywords:** Pregnancy, Childbirth, Neuroimaging, Brain tissue volumes

## Abstract

The peripartum period is the highest risk interval for the onset or exacerbation of psychiatric illness in women’s lives. Notably, pregnancy and childbirth have been associated with short-term structural and functional changes in the maternal human brain. Yet the long-term effects of pregnancy on maternal brain structure remain unknown. We investigated a large population-based cohort to examine the association between parity and brain structure. In total, 2,835 women (mean age 65.2 years; all free from dementia, stroke, and cortical brain infarcts) from the Rotterdam Study underwent magnetic resonance imaging (1.5 T) between 2005 and 2015. Associations of parity with global and lobar brain tissue volumes, white matter microstructure, and markers of vascular brain disease were examined using regression models. We found that parity was associated with a larger global gray matter volume (β = 0.14, 95% CI = 0.09–0.19), a finding that persisted following adjustment for sociodemographic factors. A non-significant dose-dependent relationship was observed between a higher number of childbirths and larger gray matter volume. The gray matter volume association with parity was globally proportional across lobes. No associations were found regarding white matter volume or integrity, nor with markers of cerebral small vessel disease. The current findings suggest that pregnancy and childbirth are associated with robust long-term changes in brain structure involving a larger global gray matter volume that persists for decades. Future studies are warranted to further investigate the mechanism and physiological relevance of these differences in brain morphology.

**Supplementary Information:**

The online version contains supplementary material available at 10.1007/s10654-021-00818-5.

## Introduction

Pregnancy and childbirth are remarkable life-changing events, from personal, social, and biological perspectives. Motherhood is an extensive adaptation, altering behavior, motivation, and emotion in the service of offspring care. Such peripartum changes in brain function have been postulated as homeostatic mechanisms to mitigate the substantially elevated risk for the onset or exacerbation of psychiatric disorders in the postpartum period [1–5].

Apart from functional changes, there is growing evidence that the maternal brain exhibits considerable structural plasticity in association with pregnancy and parturition [6–8]. Studies using animal models provide evidence that pregnancy and parturition induce profound neurobiological changes on the maternal brain in rodents [9–12]. However, few brain imaging studies have been performed to examine the structural brain changes that occur in women during pregnancy and the postpartum period. The few that do exist concluded that pregnancy is associated with a reduction of gray matter volume [13, 14]. Hoekzema and collegues [14] found that the observed postpartum reductions in specific regions of cortical gray matter remained evident two years after childbirth, suggesting that pregnancy can exert enduring structural changes on the human maternal brain. However, a study by Oatridge [13] following a small sample of healthy pregnant women serving as a control group for women with pre-eclampsia throughout pregnancy and the early postpartum period showed that global brain volume decreased during pregnancy with a nadir around the time of delivery, followed by a return to pre-pregnancy global brain volume within six months postpartum. Additional support for increasing brain size during the early postpartum period comes from a longitudinal within-subject analysis comparing images acquired 2–4 weeks postpartum with those acquired 3–4 months postpartum, which demonstrated increases of cortical gray matter volume in multiple brain regions [15]. A more recent study applied a machine learning algorithm to a longitudinal within-subject brain imaging dataset to estimate brain age in the first 2 days postpartum and again at 4–6 weeks postpartum, which revealed a “younger” brain age at 4–6 weeks postpartum [16].

Taken together, the currently available data suggest a robust morphological plasticity of the human maternal brain during pregnancy and the early postpartum period. These changes are considered to be adaptive and essential for the mother-infant bonding and sensitive caregiving. However due to relatively short and differential postpartum periods in earlier studies, the nature of the relationship between pregnancy and childbirth with brain structure remains unresolved. Considering mounting evidence of the effects of neuroendocrine physiology on brain structure and function [17, 18], it is plausible that pregnancy and childbirth might present a critical yet understudied factor that is crucial for understanding the brain processes over the life course. However, the impact of parenting behavior on the brain is also credible. Parenting is recognized to affect brain development of the offspring [19], necessitating flexibility, skill acquisition and adaptation to environment, and is reciprocally affected by the inputs from the offspring [15, 20, 21].

Therefore, we utilized data from the population-based Rotterdam Study cohort to investigate the long-term association of parity with global brain structure and vascular integrity. Advances in Magnetic resonance imaging (MRI) including Diffusion tensor imaging (DTI) methods allowed us to study the brain by integrating macrostructural morphological findings with information regarding white matter microstructural integrity. Therefore, we also investigated DTI metrics of white matter to explore if parity related to more subtle structural changes preceding macroscopic focal or global vascular alterations. Further, considering the relationship between parity and coronary heart disease and hemorrhage stroke [22, 23], we also examined white matter lesions, brain infarcts and microbleeds which are considered to be potentially important imaging markers in relation to parity.

## Methods and materials

### Setting and participants

The Rotterdam Study, a prospective population-based cohort study, includes approximately 18,000 participants aged 40 years and older living in Ommoord, a suburb of Rotterdam [24]. Since its inception in 1990, participants have undergone follow-up visits to the research center at 3–6 year intervals. At interview female participants were asked about their reproductive status including parturition history. A dedicated Magnetic resonance (MR) imaging scanner, with a fixed brain MRI protocol, was added in 2005 to the core study protocol (the Rotterdam Scan Study) [25]. Since brain MRI was introduced, more than 12,000 scans in 5913 individuals have been acquired and processed, of which 3257 are women. Of this sample, 3197 women have imaging data and information on childbirth, of which 309 had dementia or a stroke at scan date and an additional 54 had cortical infarcts on brain MRI. The final cohort included 2834 stroke-, dementia and cortical infarct-free women.

The Rotterdam Study has been approved by the Medical Ethics Committee of the Erasmus MC (registration number MEC 02.1015) and by the Dutch Ministry of Health, Welfare, and Sport (Population Screening Act WBO, license number 1071272–159,521-PG). The Rotterdam Study has been entered into the Netherlands National Trial Register (NTR; www.trialregister.nl) and into the WHO International Clinical Trials Registry Platform (ICTRP; www.who.int/ictrp/network/primary/en/) under shared catalog number NTR6831. All participants provided written informed consent to participate in the study and to have their information obtained information from their treating physicians.

### Sociodemographic, pregnancy and parity characteristics

At study entry, women were asked by trained interviewers regarding data on parity and maternal age at first childbirth, as well as sociodemographic factors. We defined parity as the number of pregnancies with a gestational age at delivery of over 24 weeks. Level of education was defined categorically as either primary education, lower general and vocational education, intermediate/higher general and intermediate vocational education, or higher vocational education/ university. Smoking history was categorized as current, former, or never. Marital history was categorized as never- or ever-married. Information on Body mass index (BMI) was obtained through interview and physical examination at a center visit closest in date to the MRI acquisition [26] and was calculated as weight (kg)/height^2^(m).

From January 2012 onwards, an additional questionnaire was added to the study protocol regarding having experienced complications during pregnancies. This question included history of any clinically diagnosed pregnancy complication, including gestational diabetes, high blood pressure during pregnancy, and pre-eclampsia, eclampsia or Hemolysis, elevated liver enzymes, and low platelet (HELLP) syndrome. This questionnaire was only available in a selected sample (894 of the women who had pregnancy information) as women who in the previous visits before 2012 had indicated they have already gone through menopause were not asked to participate in the reproductive questionnaire, including the new set of questions on pregnancy complications.

### MRI acquisition

MR images were acquired on a 1.5 T MRI scanner with an 8-channel head coil (GE signa Excite, General Electric Healthcare, Milwaukee, USA). The sequences acquired included a T1-weighted (T1w), proton density-weighted (PDw) and a fluid inversion recovery (FLAIR), all of which were used for automated brain tissue segmentation. Additionally, a T2*-weighted sequence was acquired. Full information on the scan parameters has been described previously [25]. Diffusion imaging was performed via an echo planar imaging (EPI) readout with gradients (b = 1,000 s/mm^2^) applied in 25 directions. Diffusion data were processed using a standardized pipeline, as described previously [25]. The DTI metrics included mean diffusivity (MD) which describes the rotationally invariant magnitude of water diffusion within brain tissue and fractional Anisotropy (FA), which quantifies the degree of diffusion directionality. Higher MD and lower FA (restricted to 0–1) are thought to reflect a less organized white matter architecture.

The field of view of scanning in the Rotterdam Study is focused at the supratentorial brain tissue, hence the cerebellum parenchyma was not completely included in all subjects and therefore excluded from tissue segmentation. Supratentorial tissue segmentation was performed using an automated processing algorithm based on a k-nearest-neighbour-classifier on the T1w and PDw scans complemented with FLAIR-intensity based white matter hyperintensity detection. The images were segmented into gray matter, cerebrospinal fluid, normal-appearing white matter (NAWM) and white matter hyperintensities [26, 27]. Tissue segmentation results for white matter hyperintensities and NAWM were combined with the diffusion maps to extract global measures of fractional anisotropy and mean diffusivity within NAWM. Intracranial volume (ICV) was computed by the sum of all supratentorial brain tissue classes and cerebrospinal fluid. Segmentation of the lobar regions, temporal-, frontal-, parietal- and occipital lobes, were carried out with left and right hemisphere volumes averaged for the analysis [28]. All segmentations were visually inspected by trained raters and corrected manually when needed [25, 28].

### Infarct and microbleed rating

Scans were visually inspected for the presence of lacunar infarcts and cerebral microbleeds by trained research physicians. Lacunar infarcts were defined on T2-weighted images as focal hyperintensities equal or larger than 3 mm, but smaller than 15 mm in size, and exhibiting the same characteristics as cerebrospinal fluid on all sequences–a hyperintense rim on the FLAIR sequence when located supratentorially with no involvement of the cortical gray matter. Microbleeds were defined as focal, small, round to ovoid areas of signal loss on T2*-weighted images, as previously defined [25].

### Statistical analysis

Regression analysis was performed with nulliparous/parous and parity groups (primiparous, 2–3 term pregnancies, and 4 + term pregnancies), with nulliparous women (parity = 0) as a reference in relation to imaging markers. Additionally, a stratified subgroup analysis was performed between 3 groups: nulliparous, parous women without complications during pregnancy, and parous women with complications during pregnancy. White matter hyperintensity volume was log-transformed due to a skewed distribution All variables except lacunar infarcts and microbleeds were *z*-transformed for comparison. Model I adjusted for age, and ICV. Model II further adjusted for education, marital status, smoking and body mass index. Analyses with DTI parameters as outcome were further adjusted for white matter volume and log-transformed white matter lesion volume in both models I and II.

#### Sensitivity analyses

Considering advanced age of the cohort and the suggested independent relationships of pre-eclampsia, menopause and Hormone replacement therapy (HRT) with brain structure and function [17, 18, 29, 30] we conducted two sensitivity analyses to examine whether the results of the potential effect of pregnancy on brain structure were altered by pregnancy-related complications and menopause and hormone replacement therapy. The first analysis was restricted to a subset of 894 women for whom the additional information on pregnancy complications was available. Of these, 230 women reported having experienced complications such as including gestational diabetes, high blood pressure during pregnancy, and pre-eclampsia, eclampsia or HELLP syndrome, during pregnancy.

The second analysis was conducted in a subset of 1529 women for whom menopause and HRT data were available. Details of menopause and use of hormone replacement therapy were obtained by self-report during a home interview by trained interviewers and verified through general practitioner records. Women in this subset were classified based on menopause status and use of HRT (premenopausal [*n* = 385], post-menopausal [*n* = 830], post-menopausal + HRT [*n* = 314]).

## Results

In total, 2,834 women were included in the analyses. Of these, 441 were nulliparous (parity = 0) and 2393 were parous (parity >  = 1). Further categorizing the group of parous women, 474 women were primiparous, 1697 women had 2–3 pregnancies, and 222 women had 4 or more pregnancies. The sample characteristics classified by parity are shown in Table 1.

**Table 1:** Group characteristics

	Nulliparous	Primiparous	Multiparous (parity = 2–3)	Multiparous (parity ≥ 4)
Number, n	441	474	1,697	222
Characteristics				
Age at time of MRI, years	64.45 (11.51)	62.93 (9.81)	64.20 (10.42)	69.08 (12.25)
Age at first child, years	–	27.56 (5.4)	25.23 (4)	23.96 (3.51)
Married, ever. % (n)	72 (288)	97 (415)	99 (1555)	99 (199)
Education, % (n)				
0	8 (35)	12 (57)	9 (148)	14 (30)
1	38 (168)	52 (245)	50 (854)	43 (96)
2	30 (131)	19 (92)	24 (405)	31 (69)
3	24 (105)	17 (77)	17 (284)	13 (28)
Body mass index, kg/m^2^	26.64 (4.83)	27.03 (4.70)	27.56 (4.52)	28.05 (4.73)
Smoking, n				
Never	33 (146)	37 (177)	40 (684)	51 (114)
Past smoking	47 (208)	40 (191)	44 (743)	34 (76)
Current smoking	20 (87)	22 (103)	16 (264)	13 (29)
Brain volumes				
Total brain volume, ml	905.99 (81.46)	902.65 (83.42)	900.88 (81.86)	883.45 (94.44)
Gray matter volume, ml	505.65 (45.92)	506.69 (43.09)	507.93 (44.91)	500.07 (50.20)
White matter volume, ml	393.22 (50.69)	389.59 (52.46)	387.05 (52.91)	375.15 (10.40)
Frontal lobe volume, ml	85.48 (8.69)	85.65 (8.43)	85.86 (8.60)	84.27 (9.87)
Temporal lobe volume, ml	58.81 (5.19)	59.07 (5.05)	59.00 (5.11)	57.75 (5.62)
Occipital lobe volume, ml	31.66 (3.61)	31.87 (3.25)	31.93 (3.41)	31.27 (3.52)
Parietal lobe volume, ml	49.92 (5.30)	49.89 (4.85)	50.16 (5.15)	49.63 (5.60)
Brain Microstructure				
Fractional anisotropy	0.34 (0.02)	0.34 (0.02)	0.34 (0.01)	0.34 (0.02)
Mean diffusivity, 10^–3^ mm^2^/s	0.75 (0.03)	0.74 (0.03)	0.74 (0.03)	0.76 (0.04)
Markers of cerebral small vessel disease				
White matter hyperintensity volume, ml	7.12 (13.18)	6.37 (11.59)	5.90 (8.47)	8.23 (10.40)
Lacunar infarct (Y/N)	4%	6%	6%	7%
Microbleed (Y/N)	20%	17%	17%	29%

Values are reported as means (standard deviations) unless stated otherwise. Missing values were present in education (65), marital status (243), smoking (16). Education: (0) primary education, (1) Lower general and vocational education, (2) Intermediate/higher general and intermediate vocational education, (3) higher vocational education/ university

With respect to brain tissue volumes, we found that parity was associated with a larger global gray matter volume [adjusted mean difference (β) = 0.14, 95% confidence intervals (CI) = 0.09;0.19] (Tables 2, 3). This association persisted following adjustment for smoking, BMI, education, and history of marital status (β = 0.10, 95% CI = 0.04;0.17). We found no differences in white matter volume associated with parity. The relationship between gray matter volume and parity was consistent across temporal, frontal, occipital and parietal regions (Supplementary Table 1, Model I). These relationships were attenuated after adjustment for sociodemographic factors (Supplementary Table 1, Model II). No disproportionate lobar changes were found.

**Table 2:** Relationship between parity (parous/nulliparous) and structural brain imaging markers

	Model I	Model II
	Beta (95% CI)	Beta (95% CI)
Total brain volume	0.08 (0.04;0.12)	0.07 (0.03;0.12)
Gray matter volume	0.14 (0.09;0.19)	0.11 (0.04;0.17)
White matter volume	0.00 (− 0.05;0.06)	0.02 (− 0.04;0.09)
Fractional anisotropy	0.05 (− 0.04;0.15)	0.03 (− 0.08;0.14)
Mean Diffusivity	− 0.07 (− 0.14;0.00)	− 0.06 (− 0.14;0.02)
White matter hyperintensity volume	− 0.01 (− 0.09;0.06)	− 0.00 (− 0.08;0.08)
Lacunar Infarct	0.02 (− 0.01;0.04)	0.01 (− 0.01;0.04)
Microbleed	− 0.01 (− 0.05;0.03)	0.01 (− 0.04;0.05)

White matter lesion volume was log-transformed due to a skewed distribution. All variables except infarct and microbleeds were *z*-transformed to allow for comparison. Bold indicates *p* < 0.05

Model I was adjusted for age and intracranial volume. Model II further adjusted for education, body mass index, smoking and marital history. DTI analysis further adjusted for white matter volume and white matter lesion volume in both models I and II

**Table 3:** Relationship between the parity and structural brain imaging markers

Nulliparous as reference	Primiparous	Multiparous (parity = 2–3)	Multiparous (parity ≥ 4)
	Beta (95% CI)	Beta (95% CI)	Beta (95% CI)
Model I			
Total brain volume	**0.07(0.02;0.12)**	**0.09(0.05;0.13)**	**0.07 (0.01;0.13)**
Gray matter volume	**0.10 (0.03;0.17)**	**0.15 (0.09;0.21)**	**0.15 (0.06;0.24)**
White matter volume	0.01 (− 0.06;0.08)	0 (− 0.06;0.06)	− 0.02 (− 0.11;0.07)
Fractional anisotropy	0 (− 0.12;0.12)	0.08 (− 0.01;0.17)	− 0.01 (− 0.16;0.13)
Mean Diffusivity	**− 0.08 (− 0.17;0.00)**	**− 0.08 (− 0.15;− 0.01)**	0.00 (− 0.10;0.11)
White matter hyperintensity volume	0.03 (− 0.06;0.12)	− 0.03 (− 0.1;0.05)	− 0.01 (− 0.12;0.11)
Lacunar Infarct	0.02 (− 0.01;0.05)	0.02 (− 0.01;0.04)	0.02 (− 0.02;0.05)
Microbleed	− 0.01 (− 0.06;0.04)	− 0.02 (− 0.06;0.02)	0.05 (− 0.01;0.12)
Model II			
Total brain volume	**0.06(0.01;0.11)**	**0.07 (0.03;0.12)**	**0.09 (0.02;0.15)**
Gray matter volume	**0.08 (0.00;0.16)**	**0.11 (0.05;0.18)**	**0.13 (0.03;0.22)**
White matter volume	0.02 (− 0.06;0.10)	0.02 (− 0.05;0.09)	0.02 (− 0.07;0.12)
Fractional anisotropy	− 0.04 (− 0.17;0.09)	0.06 (− 0.05;0.17)	− 0.03 (− 0.19;0.13)
Mean Diffusivity	− 0.07 (− 0.17;0.03)	− 0.06 (− 0.15;0.02)	0.01 (− 0.11;0.13)
White matter hyperintensity volume	0.02 (− 0.08;0.12)	− 0.01 (− 0.09;0.08)	0.02 (− 0.11;0.14)
Lacunar Infarct	0.02 (− 0.01;0.06)	0.01 (− 0.02;0.04)	0.01 (− 0.03;0.05)
Microbleed	0.00 (− 0.06;0.06)	0.00 (− 0.05;0.05)	**0.08(0.01;0.14)**

White matter lesion volume was log-transformed due to a skewed distribution All variables except infarct and microbleeds were *z*-transformed to allow for comparison. Bold indicates *p* < 0.05

Model I was adjusted for age and intracranial volume. Model II further adjusted for education, body mass index, smoking and marital history. DTI analysis further adjusted for white matter volume and white matter lesion volume in both models I and II

For analyses involving microstructural outcomes, mean diffusivity was lower in parous women (β = − 0.07, 95% CI = − 0.14;0.00). No relationships were observed between parity and fractional anisotropy in normal-appearing white matter (NAWM). There was also no relationship between parity and markers of cerebral small vessel disease, with the exception of an increase of microbleeds observed in multiparous (parity ≥ 4) women compared to nulliparous women [β = 0.07, 95% CI = 0.01;0.13] (Table 3, Model II).

We next examined whether the association between parity and brain structure might be differentially influenced by parity. Nulliparous women were considered as the reference group. The results showed a perceived incremental increase in the association between parity and gray-matter volume (Table 3, Model I in reference to nulliparous women; primiparous [β = 0.10, 95% CI = 0.03;0.17], multiparous women (parity: 2–3) [β = 0.15, 95% CI = 0.09;0.21], multiparous women (parity ≥ 4) [β = 0.15, 95% CI = 0.06;0.24]). However, there is no significant difference between primiparous and multiparous women (2–3, and 4 +). Furthermore, these associations attenuated after adjustment for sociodemographic factors (Table 3, Model II; primiparous [β = 0.07, 95% CI = ¬ 0.00;0.15], multiparous (parity = 2–3) [β = 0.12, 95% CI = 0.05;0.18], multiparous (parity ≥ 4) [β = 0.12, 95% CI = 0.02;0.22]). No other brain imaging marker studied exhibited an increase in association strength with respect to parity (Table 3).

### Sensitivity analysis

In total, 894 women had information on pregnancy-related complications. Of these, 664 women reported no complications, and 230 women reported a history of complications during pregnancy. The subsample characteristics are shown in Table 4, stratified by pregnancy complications (parous without pregnancy complications, parous with pregnancy complications).

**Table 4:** Pregnancy-related complications: group characteristics

	Parous without complications during pregnancy	Parous with complications during pregnancy
Number, n	664	230
Characteristics		
Age at MRI, years; mean (SD)	57.51 (6.48)	57.65 (6.02)
Age at first child, years	25.88 (4.70)	26.04 (4.67)
Number of children	2.08 (0.94)	2.13 (0.81)
Married % (n)	98 (650)	100 (230)
Education % (n)		
0	10 (67)	6 (14)
1	42 (276)	44 (102)
2	23 (155)	24 (56)
3	25 (165)	25 (57)
Body mass index, kg/m2	26.89 (4.29)	28.33 (5.48)
Smoking, n		
Never	35 (230)	40 (91)
Past smoking	43 (287)	43 (100)
Current smoking	22 (146)	17 (38)
Brain Volumes		
Total brain volume, ml	927.18 (79.52)	922.45 (82.96)
Gray matter volume, ml	517.69 (46.24)	514.71 (43.67)
White matter volume, ml	401.11 (49.96)	404.43 (52.45)
Frontal lobe volume, ml	88.08 (8,70)	87.74 (8.42)
Temporal lobe volume, ml	60,31 (5.22)	59.83 (5.02)
Occipital lobe volume, ml	32.65 (3.46)	32.54 (3.20)
Parietal lobe volume, ml	50.96 (5.38)	50.55 (4.88)
Brain Microstructure		
Fractional anisotropy	0.33 (0.01)	0.33 (0.01)
Mean diffusivity, 10–3 mm2/s	0.74 (0.02)	0.73 (0.02)
Markers of cerebral small vessel disease		
White matter hyperintensity volume, ml	3.22 (4.46)	3.31 (6.01)
Infarct (Y/N)	3%	4%
Microbleed (Y/N)	12%	13%

Values are reported as means (standard deviations) unless stated otherwise. Education; (0) primary education, (1) Lower general and vocational education, (2) Intermediate/higher general and intermediate vocational education, (3) higher vocational education/ university. Missing from education and smoking (*n* = 1 both groups)

Evaluation of the subsample of women for whom information on pregnancy-related complications was available yielded differences exclusively in white matter between women with complications during pregnancy compared to parous women without pregnancy-related complications. Women with pregnancy-related complications exhibited larger white matter volumes (Table 5, Model I; β = 0.08, 95% CI = 0.01;0.16). This relationship attenuated after adjustment for smoking, BMI, and education (Table 5). Analysis of the relationship of parity with gray matter volume and MD showed similar effects as in the overall sample (Table 5, Model 1).

**Table 5:** Relationship between pregnancy complications and parity and structural brain imaging markers

	Parous with complications -Parous without complications during pregnancy (ref)	Parous without complications during pregnancy -Nulliparous (ref)	Parous with complications during pregnancy -Nulliparous (ref)
	Beta (95% CI)	Beta (95% CI)	Beta (95% CI)
Model I			
Total brain volume	− 0.04(− 0.01;0.08)	**0.11(0.07;0.16)**	**0.15(0.09;0.21)**
Gray matter volume	− 0.02 (− 0.11;− 0.05)	**0.14 (0.07;0.21)**	**0.11 (0.02;0.20)**
White matter volume	**0.08 (0.01;0.16)**	0.05 (− 0.02;0.012)	**0.13 (0.05;0.22)**
Fractional anisotropy	− 0.04 (− 0.17;0.09)	0.04 (− 0.15;0.08)	− 0.08 (− 0.23;0.07)
Mean diffusivity	− 0.02 (− 0.11;0.06)	**− 0.13 (− 0.21;-0.04)**	**− 0.15 (− 0.26;-0.04)**
White matter hyperintensity volume	0.06 (− 0.03;0.15)	**− 0.09 (− 0.18;0.00)**	− 0.02 (− 0.14;0.09)
Lacunar Infarct	0.00 (− 0.02;0.03)	0.01 (− 0.01;0.04)	0.01 (− 0.02;0.05)
Microbleed	0.00 (− 0.04;0.05)	− 0.02 (− 0.07;0.02)	− 0.02 (− 0.08;0.04)
Model II			
Total brain volume	0.04(− 0.01;0.09)	**0.09(0.04;0.14)**	**0.12(0.06;0.19)**
Gray matter volume	− 0.01 (− 0. 09;0.08)	**0.09 (0.01;0.17)**	0.07 (− 0.03;0.16)
White matter volume	0.07 (− 0.01;0.15)	− 0.07 (− 0.01;0.15)	**0.13 (0.04;0.23)**
Fractional anisotropy	− 0.02 (− 0.15;0.11)	− 0.08 (− 0.21;0.04)	− 0.09 (− 0.26;0.07)
Mean diffusivity	− 0.03 (− 0.12;0.06)	− 0.08 (− 0.18;0.00)	**− 0.12 (− 0.25;0.00)**
White matter hyperintensity volume	0.01 (− 0.08;0.11)	− 0.03 (− 0.12;0.07)	0.01 (− 0.12;0.13)
Lacunar Infarct	0.00 (− 0.02;0.03)	0.01 (− 0.02;0.03)	0.01 (− 0.03;0.04)
Microbleed	0.01 (−0.05;0.06)	− 0.02 (− 0.07;0.03)	0.01 (− 0.06;0.19)

White matter lesion volume was log-transformed due to a skewed distribution. All variables except infarct and microbleeds were *z*-transformed to allow for comparison. Bold indicates *p* < 0.05. Model I was adjusted for age and intracranial volume. Model II further adjusted for education, body mass index, smoking and marital history. DTI analysis further adjusted for white matter volume and white matter lesion volume in both models I and II. Ref; reference variable

A sensitivity analysis for the influence of menopause status and HRT on the relationship between parity and larger gray matter volume yielded no significant effects (β = 0.10, 95% CI = 0.02;0.17) (Supplementary Table 2).

## Discussion

This population-based study observed an association between parity and brain structure decades following pregnancy and childbirth. Specifically, we found that parity was associated with larger total gray matter volume later in life, a finding that persisted following adjustment for sociodemographic factors. The larger gray matter volume associated with parity appeared not to be driven by specific lobar brain regions, but rather was globally proportional across lobes. Further, the analysis revealed lower MD in relation to pregnancy suggesting healthier white matter status, however the observed effect was significantly reduced when adjusting for psychosocial factors. Moreover, we did not find evidence for an association between parity and markers of cerebral small vessel disease, which supports the theory of the adaptation of the brain and cerebral circulation during pregnancy to maintain brain homeostasis, despite substantial peripartum hormonal and cardiovascular changes [29].

A recently published study suggested that pregnancy-induced reductions in gray matter remained evident for at least two years after childbirth, implying a long-term reduction in brain tissue volume, primarily located in specific lobe regions [14]. Here we found that decades after pregnancy, gray matter volume is actually larger, a finding that remained robust following adjustment for age, BMI, smoking, education, and marital status [31, 32]. Moreover, similar association of parity and gray matter volume was also found in women who have experienced pregnancy-related complications. Despite extensive reports on associations between pre-eclampsia and changes in cortical volumes [13, 33–35], this finding is in line with a prior study reporting no influence of preeclampsia on the association of parity and global gray matter volume in the early postpartum period [13].

Surprisingly, white matter volume was larger in women with pregnancy-related complications versus nulliparous women and compared to women without pregnancy-related complications. A possible link underlying the association of larger white matter volume and hypertension-related complications might relate to a homeostatic compensation for chronic vascular insufficiency. However, there is also a possibility that acute vascular events early in adulthood might have led to compensatory mechanisms later in life, which increased neuroplasticity or reduced white matter degeneration. Finally, we cannot discard the possibility of a chance finding. We also acknowledge that exclusion of women who suffered from stroke, dementia, or cortical infarct might have introduced a selection bias. However, our decision to exclude those women was predicated upon the reliability of the (automated segmentation of) imaging markers.

There is a consensus that pregnancy, delivery, and puerperium expose women to a diversity of health changes that extend beyond direct obstetric complications of pregnancy. For example, emerging evidence suggests that women in the early postpartum period have a substantially increased risk of a first-onset or exacerbation of psychiatric disorders, cardiovascular, and autoimmune diseases [36–38]. However, investigations of the long-term risks associated with pregnancy and delivery have been inconclusive [39–42]. While some studies have argued that childbirth is associated with accelerated cellular aging due to higher levels of oxidative stress [39], other studies have contradicted these findings by demonstrating elongated telomeres suggestive of an attenuation of risk [40, 43]. Although no unifying biological explanation has emerged to explain these apparently contrasting findings, it remains a distinct possibility that pregnancy and childbirth have an enduring influence on the endocrine system, and consequently on brain structure, long after childbirth. Pregnancy and childbirth are accompanied by dramatic changes in the hormonal profile. Prolactin, androgens, and estrogens exhibit multiple orders of magnitude increases that are thought to create an anti-inflammatory environment to support pregnancy, fetal growth, and delivery [37, 44, 45]. For example, during pregnancy increased levels of progesterone stimulate the differentiation of T cells into T-helper type 2 cells, which release anti-inflammatory cytokines [46]. Circulating estradiol also functions during pregnancy in the mediation and control of immunosuppressive regulatory B cells [47]. Hormonal fluctuations and their interactions with immune processes have been suggested to modulate several forms of brain plasticity, including changes in glial proliferation, neuronal morphology, and neurogenesis [48, 49], however their long-term effects on brain aging are not completely understood.

Another potential explanation of enduring effects of pregnancy on brain structure is the bi-directional trafficking of maternal and fetal cells throughout gestation, which can acquire long-term residence in the human brain [50–56]. Fetal cells have been found at the sites of inflammation and linked to preeclampsia and multiple autoimmune diseases [54, 57]. Inflammation has also been associated with structural and functional brain changes [57]. Fetal cells are able to integrate into maternal brain circuity and express appropriate immunochemical markers for brain tissues [51, 53]. However, the extent to which fetal microchimerism is tolerated and whether dynamic changes occur over time remain unknown.

In parallel with underlying biological mechanisms of the observed association between parity and brain structure, the experience of parenting may also alter the brain, which is assumed to be necessary to support sensitive and responsive caregiving. A small number of studies have reported increased gray matter volume of the prefrontal cortex and in parietal lobes. Also, functional brain changes such as higher activation in superior temporal sulcus and amygdala activation were reported to be associated with parenthood in humans [15, 20, 58]. Further, it has been shown that the duration of motherhood is associated with greater neural activation to infant-specific cues [21, 59]. Another study found that foster mothers demonstrated an association between brain activity and caregiving behavior comparable to the associations observed in biological mothers [60]. Furthermore, a recent neuroimaging study in older adults found a positive association between the number of offspring and cortical thickness, in both fathers and mothers [61].

Our study has several limitations. Although data were sampled from a large, prospective, longitudinal population-based study allowing us to adjust for several covariates, we did not have information on infertility and gravidity which might have improved our ability to adjust for potential confounders. The limited available data did not allow us to explore the mechanistic pathways fully through more advanced methodologies such as directed acyclic graph. Hence, this study utilized a cross-sectional design, which precluded firm conclusions regarding the causality of the observed results. Furthermore, availability of time-varying cardiovascular risk factors might have been helpful to assess the mediating effects on the relationships between parity and brain volumes. The small sample of women with pregnancy-related complications restricted our ability to make distinctions among the various specific complications, which might have further clarified the observed results. Additionally, our sample consisted of a predominantly middle-class population of Caucasian descent, which may restrict the generalizability of our findings. Also, although structural volume segmentation at 1.5 T at the aggregated level we used is likely highly comparable to 3 T field strength, more in-depth investigations may benefit from higher resolution and/or higher field strength imaging [62].

Moreover, information regarding pregnancy complications were available in a smaller subset of women from whom ~ 25% reported having experienced any pregnancy-related complications, which is larger than prior prevalence estimates of pregnancy-related complications [63–65]. As this questionnaire–at its introduction in the Rotterdam Study–was not asked from all women, the possibility of selection bias cannot be entirely ruled out. Moreover, the data was acquired using a self-report questionnaire. Considering no external validation data such as use of medication or treatment for pregnancy complications was available [66], recall bias cannot be excluded.

In addition, we acknowledge that we cannot distinguish between the effects of pregnancy, parity, and parenting on structural changes of the brain. Furthermore, it is possible that the observed effect of parity is a result of smaller brain volumes among nulliparous women, rather than larger gray matter volume in parous women. The important point in this context is that nulliparous and parous women might differ in several ways regarding their partnerships and unplanned pregnancies. Living in a relationship with a partner might have cognitive and social challenges that result in enduring changes of brain volume. Although we adjusted for the history of marital status, we cannot rule out residual confounding by factors such as unregistered partnerships. Hence, while speculative, considering the study design and advanced age of the cohort, the findings may be interpreted in several ways. One possible interpretation is that over the life course, pregnancy and childbirth lead to an increase in global gray matter volume. Alternatively, pregnancy and childbirth may serve as a protective factor for subsequent age-related brain atrophy. It is also possible that women who pursue motherhood might differ in their brain structure from those who do not. Lastly, it might be that parenting creates an enriched social network that is protective against brain ageing.

In conclusion, the current findings indicate that parity is associated with a relatively larger global gray matter volume, decades following childbirth. Although the mechanism and physiological relevance of the morphological alterations remain unknown, these data provide novel insight into the long-term impact of motherhood on the human brain.

## Supplementary Information

Below is the link to the electronic supplementary material.

Supplementary file1 (DOCX 20 kb)
