# Supplementary material for: Long-term association of pregnancy and maternal brain structure: the Rotterdam Study
Source: Eur J Epidemiol. 2022 Jan 6;37(3):271–81. doi: 10.1007/s10654-021-00818-5 (PMC9110529; doi:10.1007/s10654-021-00818-5)
Supplement: Supplementary file 1 — Supplementary file1 (DOCX 20 kb) [file 10654_2021_818_MOESM1_ESM.docx]

**SUPPLEMENT**

**Supplementary Table 1**. Relationship between parity and gray matter volumes

|  | primiparous-nulliparous | Multiparous  (parity =2-3)-  nulliparous | Multiparous  (parity ≥4)-nulliparous |
| --- | --- | --- | --- |
|  | Beta (95% CI) | Beta (95% CI) | Beta (95% CI) |
| ***Model I*** |  |  |  |
| Total | **0.10 (0.03;0.17)** | **0.15 (0.09;0.21)** | **0.15 (0.06;0.24)** |
| Frontal lobe | **0.09 (0.01;0.16)** | **0.14 (0.08;0.20)** | **0.16 (0.07;0.25)** |
| Temporal lobe | **0.12 (0.04;0.19)** | **0.13 (0.07;0.19)** | 0.09 (-0.01;0.18) |
| Occipital lobe | **0.12 (0.03;0.21)** | **0.16 (0.09;0.23)** | **0.14 (0.03;0.25)** |
| Parietal lobe | **0.09 (0.01;0.17)** | **0.15 (0.08;0.21)** | **0.16 (0.07;0.26)** |
| ***Model II*** |  |  |  |
| Total | **0.08 (0.00;0.16)** | **0.11 (0.05;0.18)** | **0.13 (0.03;0.22)** |
| Frontal lobe | 0.07 (-0.01;0.15) | **0.11 (0.04;0.18)** | **0.14 (0.04;0.24)** |
| Temporal lobe | **0.10 (0.02;0.19)** | **0.10 (0.03;0.17)** | 0.07 (-0.03;0.18) |
| Occipital lobe | **0.10 (0.00;0.20)** | **0.11 (0.03;0.20)** | **0.13 (0.00;0.25)** |
| Parietal lobe | 0.05 (-0.03;0.14) | **0.11 (0.04;0.18)** | **0.14 (0.03;0.25)** |

White matter lesion volume was log-transformed due to a skewed distribution. All variables were *z*-transformed to allow for comparison. Bold indicates p<0.05. Model I was adjusted for age and intracranial volume. Model II further adjusted for education, body mass index, smoking and marital history.

|  | Model I + Menopause/HRT |
| --- | --- |
|  | Beta (95% CI) |
|  |  |
| Total brain volume | **0.11 (0.06;0.16)** |
| Gray matter volume | **0.10 (0.02;0.17)** |
| White matter volume | **0.07 (0.00;0.14)** |
|  |  |
| Fractional anisotropy | 0.03 (-0.09;0.15) |
| Mean Diffusivity | -0.04 (-0.12;0.04) |
|  |  |
| White matter hyperintensity volume | -0.01 (-0.10;0.08) |
| Lacunar Infarct | 0.01(-0.02;0.03) |
| Microbleed | -0.02 (-0.06;0.03) |

**Supplementary** **Table 2.** Relationship between the parity (parous/nulliparous) and structural brain imaging markers after adjusting for menopause status and hormone replacement therapy.

Model I adjusts for age and ICV. Additionally, menopause and hormone replacement therapy (HRT) is adjusted as a factor variable coded as 0: no menopause at scan, 1: menopause and use of HRT, 2: menopause and no use of HRT. DTI analysis further adjusted for white matter volume and white matter lesion volume.
